# Quantitative assessment of the bidirectional relationships between diabetes and depression

**DOI:** 10.18632/oncotarget.15051

**Published:** 2017-02-03

**Authors:** Qi-Shuai Zhuang, Liang Shen, Hong-Fang Ji

**Affiliations:** ^1^ Shandong Provincial Research Center for Bioinformatic Engineering and Technique, School of Life Sciences, Shandong University of Technology, Zibo, P. R. China

**Keywords:** diabetes, depression, risk, bidirectional relationships

## Abstract

Diabetes and depression impose an enormous public health burden and the present study aimed to assess quantitatively the bidirectional relationships between the two disorders. We searched databases for eligible articles published until October 2016. A total of 51 studies were finally included in the present bidirectional meta-analysis, among which, 32 studies were about the direction of depression leading to diabetes, and 24 studies about the direction of diabetes leading to depression. Pooled results of the 32 eligible studies covering 1274337 subjects showed that depression patients were at higher risk for diabetes (odds ratio (OR) = 1.34, 95% confidence intervals (CI) = [1.23, 1.46]) than non-depressive subjects. Further gender-subgroup analysis found that the strength of this relationship was stronger in men (OR = 1.63, 95%CI = [1.48, 1.78]) than in women (OR = 1.29, 95%CI = [1.07, 1.51]). For the direction of diabetes leading to depression, pooled data of 24 articles containing 329658 subjects showed that patients with diabetes were at higher risk for diabetes (OR = 1.28, 95%CI = [1.15, 1.42]) than non-diabetic subjects. The available data supports that the relationships between diabetes and depression are bidirectional and the overall strengths are similar in both directions. More mechanistic studies are encouraged to explore the molecular mechanisms underlying the relationships between the two diseases.

## INTRODUCTION

Diabetes is a chronic metabolic disease with increasing prevalence worldwide nowadays owing to the lifestyle modifications and increasing life expectancy [1]. According to the International Diabetes Federation, 415 million adults have diabetes and this figure is expected to rise to 642 million by 2040 [2]. As high blood glucose levels can damage blood vessels, nerves, eyes and kidneys, patients with diabetes may suffer from a number of serious complications. Depression is a common psychiatric mood disorder with increasing prevalence and is one of the largest single causes of disability around the world [3].

Diabetes and depression carry a large public health burden presently and also a heavy burden on health budgets in the future. In recent years, accumulating studies indicate the close associations between the two chronic diseases, diabetes and depression [4–7]. It is found that comorbid diabetes and depression is common, which results in significant detrimental impact on health outcomes, including a significantly increased risk of mortality and increased healthcare cost [8]. Although there is a high rate of comorbid depression in patients with diabetes, it is unrecognized and untreated largely [9, 10]. Moreover, the prevalence of diabetes and depression differs in men and women. Women are about twice as likely as men to develop depression, while the prevalence of diabetes is higher in men than women [11, 12]. Thus, the present study aimed to perform a bidirectional random effects meta-analysis to quantitatively assess the strength of the relationships between diabetes and depression, and to examine the difference of the strength in males and females, to provide implications for future interventional studies of the two diseases.

## RESULTS

### Risk of diabetes in patients with depression

**Figure 1:**
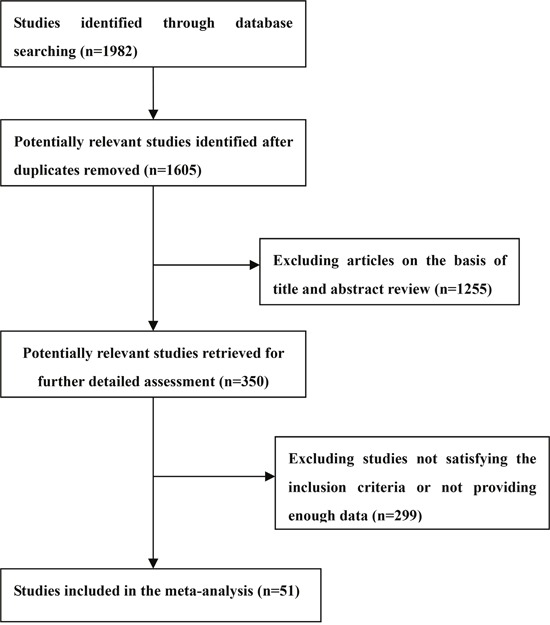
Selection of studies for inclusion in the meta-analysis

**Figure 2:**
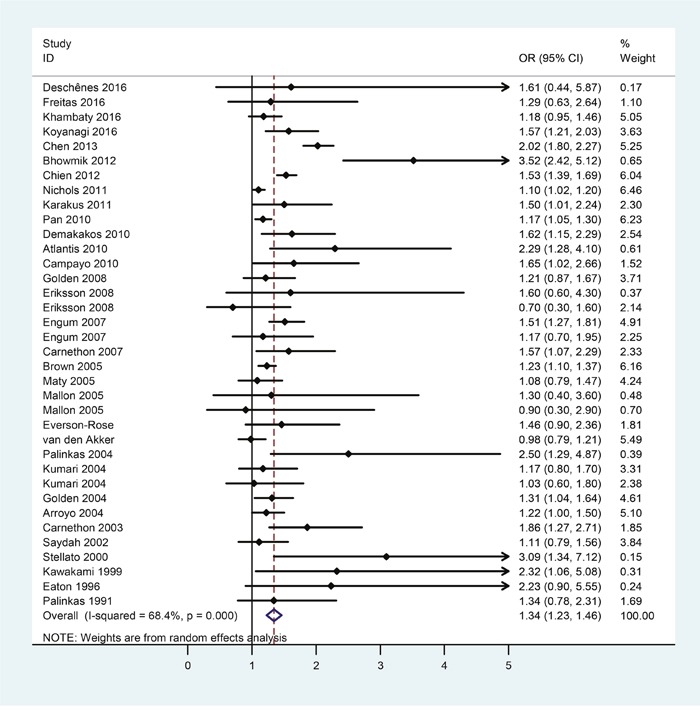
Forest plots of risk of diabetes in patients with depression

Selection process of studies included in the present study was shown in Figure 1. There were 32 eligible studies covering 1274337 subjects included in the assessment of risk of diabetes in patients with depression. Table 1 showed the first author, year of publication, country, number of subjects, the odds ratio (OR) and the corresponding 95% confidence intervals (CI), extracted from each study. The meta-analytic results were shown in Figure 2, which indicated that depression patients were at higher risk of developing diabetes (OR = 1.34, 95%CI = [1.23, 1.46]). Statistically moderate heterogeneity was found among these studies (*p* = 0.000, *I^2^* = 68.4%).

**Table 1:** Characteristics of the studies included in the analysis of risk of diabetes in patients with depression

References	Country	Subjects	Age	Weight (%)	OR	95% CI
Deschênes 2016 [13]	Canada	2486	40-69	0.17	1.61	0.44-5.87
Freitas 2016 [14]	England	4454	50+	1.10	1.29	0.63-2.64
Khambaty 2016 [15]	US	2156	60+	5.05	1.18	0.95-1.46
Koyanagi 2016 [16]	Spain	201 337	18+	3.63	1.57	1.21-2.03
Chen 2013 [17]	China	11694	60.1±13.2	5.25	2.02	1.80-2.27
Bhowmik 2012 [18]	Bangladesh	2293	20+	0.65	3.52	2.42-5.12
Chien 2012 [19]	China	766427	18+	6.04	1.53	1.39-1.69
Nichols 2011 [20]	US	58056	50+	6.46	1.10	1.02-1.20
Karakus 2011 [21]	US	12652	51-61	2.30	1.50	1.01-2.24
Pan 2010 [22]	US	65381	50-75	6.23	1.17	1.05-1.30
Demakakos 2010 [23]	UK	6111	50+	2.54	1.62	1.15-2.29
Atlantis 2010 [24]	Australia	1000	65+	0.61	2.29	1.28-4.10
Campayo 2010 [25]	Spain	3521	55+	1.52	1.65	1.02-2.66
Golden 2008 [26]	US	5201	45-84	3.71	1.21	0.87-1.67
Eriksson 2008 [27]	Sweden	2127	35+	0.37	1.60	0.60-4.30
Eriksson 2008 [27]	Sweden	3100	35+	2.14	0.70	0.30-1.60
Engum 2007 [28]	Norway	37291	30-89	4.91	1.51	1.27-1.81
Engum 2007 [28]	Norway	37291	30-89	2.25	1.17	0.70-1.95
Carnethon 2007 [29]	US	4681	65+	2.33	1.57	1.07-2.29
Brown 2005 [30]	Canada	92677	20-50	6.16	1.23	1.10-1.37
Maty 2005 [31]	US	6147	17-94	4.24	1.08	0.79-1.47
Mallon 2005 [32]	Sweden	550	45-65	0.48	1.30	0.40-3.60
Mallon 2005 [32]	Sweden	620	45-65	0.70	0.90	0.30-2.90
Everson-Rose 2004 [33]	US	2662	42-52	1.81	1.46	0.90-2.36
van den Akker 2004 [34]	Netherlands	68004	20-50	5.49	0.98	0.79-1.21
Palinkas 2004 [35]	US	971	50-89	0.39	2.50	1.29-4.87
Kumari 2004 [36]	UK	5807	35-55	3.31	1.17	0.80-1.70
Kumari 2004 [36]	UK	2579	35-55	2.38	1.03	0.60-1.80
Golden 2004 [37]	US	11615	45-64	4.61	1.31	1.04-1.64
Arroyo 2004 [38]	US	72178	45-72	5.10	1.22	1.00-1.50
Carnethon 2003 [39]	US	6190	25-74	1.85	1.86	1.27-2.71
Saydah 2002 [40]	US	8870	32-86	3.84	1.11	0.79-1.56
Stellato 2000 [41]	US	1156	40-70	0.15	3.09	1.34-7.12
Kawakami 1999 [42]	Japan	2380	18-53	0.31	2.32	1.06-5.08
Eaton 1996 [43]	US	1715	18+	0.24	2.23	0.90-5.55
Palinkas 1991 [44]	US	1585	50+	1.69	1.34	0.78-2.31

### Risk of diabetes in male patients with depression

**Figure 3:**
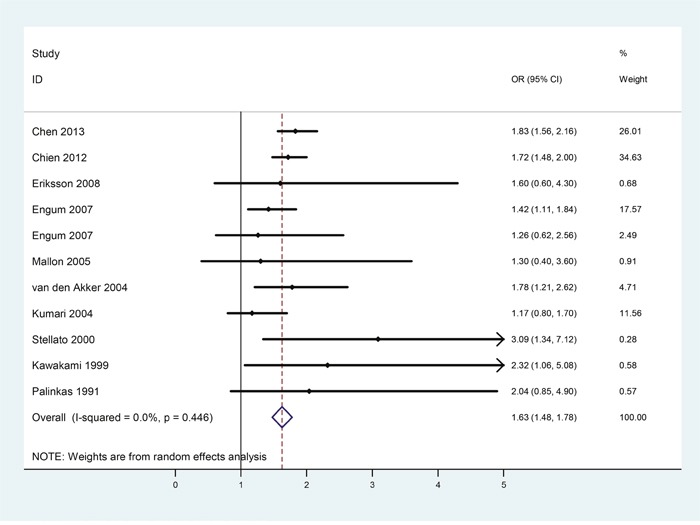
Forest plots of risk of diabetes in male patients with depression

Effects of gender on the strength of the bidirectional relationships between diabetes and depression were examined through further gender-subgroup analysis. A total of 10 studies were included in the male subgroup analysis. Table 2 listed the main characteristics about the number of male subjects, OR and the corresponding 95% CI of each study. According to the meta-analytic results shown in Figure 3, depression patients were at 63% increased risk for developing diabetes (OR = 1.63, 95%CI = [1.48, 1.78]) than non-depressive male subjects, and heterogeneity was not found among these studies (*p* = 0.446, *I^2^* = 0.0%).

**Table 2:** Characteristics of the studies included in the male subgroup analysis of risk of diabetes in patients with depression

References	Country	Subjects	Males %	Age	Weight (%)	OR	95% CI
Chen 2013 [17]	China	11694	40.3	60.1±13.2	26.01	1.83	1.56-2.16
Chien 2012 [19]	China	766427	48.8	18+	34.63	1.72	1.48-2.00
Eriksson 2008 [27]	Sweden	5227	40.7	35+	0.68	1.60	0.60-4.30
Engum 2007 [28]	Norway	37291	47.2	30-89	17.57	1.42	1.11-1.84
Engum 2007 [28]	Norway	37291	47.2	30-89	2.49	1.26	0.62-2.56
Mallon 2005 [32]	Sweden	1170	47	45-65	0.91	1.30	0.40-3.60
van den Akker 2004 [34]	Netherlands	68004	48.8	20-50	4.71	1.78	1.21-2.62
Kumari 2004 [36]	US	8386	69.2	35-55	11.56	1.17	0.80-1.70
Stellato 2000 [41]	US	1156	100	40-70	0.28	3.09	1.34-7.12
Kawakami 1999 [42]	Japan	2380	100	18-53	0.58	2.32	1.06-5.08
Palinkas 1991 [44]	US	1585	46.7	50+	0.57	2.04	0.85-4.90

### Risk of diabetes in female patients with depression

**Figure 4:**
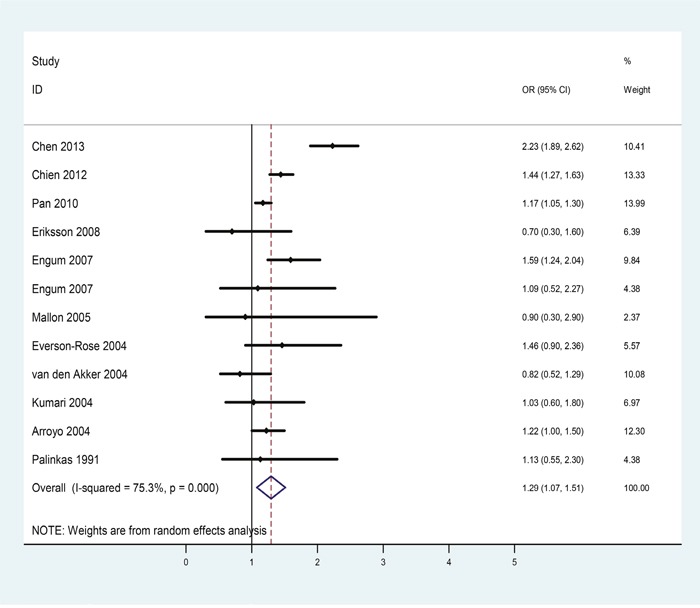
Forest plots of risk of diabetes in female patients with depression

There were 11 eligible studies included in the female subgroup analysis. Table 3 showed the extracted information of each eligible study. The pooled OR indicated that depression patients were at higher risk for diabetes (OR = 1.29, 95%CI = [1.07, 1.51], Figure 4) than non-depressive female subjects and statistically moderate heterogeneity was found among these studies (*p* = 0.000, *I^2^* = 75.3%).

**Table 3:** Characteristics of the studies included in the female subgroup analysis of risk of diabetes in patients with depression

References	Country	Subjects	Females %	Age	Weight (%)	OR	95% CI
Chen 2013 [17]	China	11694	59.7	60.1±13.2	10.41	2.23	1.89-2.62
Chien 2012 [19]	China	766427	51.2	18+	13.33	1.44	1.27-1.63
Pan 2010 [22]	US	65381	100	50-75	13.99	1.17	1.05-1.30
Eriksson 2008 [27]	Sweden	5227	59.3	35+	6.39	0.70	0.30-1.60
Engum 2007 [28]	Norway	37291	52.8	30-89	9.84	1.59	1.24-2.04
Engum 2007 [28]	Norway	37291	52.8	30-89	4.38	1.09	0.52-2.27
Mallon 2005 [32]	Sweden	1170	53	45-65	2.37	0.90	0.30-2.90
Everson-Rose 2004 [33]	US	2662	100	42-52	5.57	1.46	0.90-2.36
van den Akker 2004 [34]	Netherlands	68004	51.2	20-50	10.08	0.82	0.52-1.29
Kumari 2004 [36]	US	8386	30.8	35-55	6.97	1.03	0.60-1.80
Arroyo 2004 [38]	US	72178	100	45-72	12.30	1.22	1.00-1.50
Palinkas 1991 [44]	US	1585	53.3	50+	4.38	1.13	0.55-2.30

### Risk of depression in patients with diabetes

**Figure 5:**
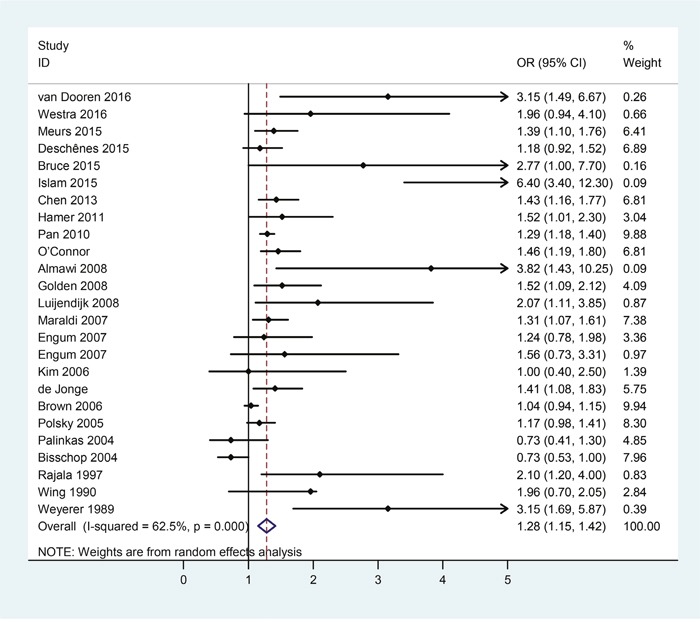
Forest plots of risk of depression in patients with diabetes

For the direction of diabetes leading to depression, there were a total of 24 eligible studies covering 329658 subjects. Table 4 collected the extracted main characteristics, including the first author, year of publication, country, and number of subjects, OR and the corresponding 95% CI of each included study. The pooled results were shown in Figure 5, which indicated that patients with diabetes were at higher risk for developing depression (OR = 1.28, 95%CI = [1.15, 1.42]) than non-diabetic subjects with statistically significant heterogeneity among these studies (*p* = 0.000, *I^2^* = 62.5%).

**Table 4:** Characteristics of the studies included in the analysis of risk of depression in patients with diabetes

References	Country	Subjects	Age	Weight (%)	OR	95% CI
van Dooren 2016 [45]	Netherlands	862	40-75	0.26	3.15	1.49-6.67
Westra 2016 [46]	Netherlands	527	60-87	0.66	1.96	0.94-4.10
Meurs 2015 [47]	Netherlands	90686	18-93	6.41	1.39	1.10-1.76
Deschênes 2015 [48]	Canada	17623	15+	6.89	1.18	0.92-1.52
Bruce 2015 [49]	Australia	184	70.2±10.1	0.16	2.77	1.00-7.70
Islam 2015 [50]	Australia	1182	50.4±11.4	0.09	6.40	3.40-12.30
Chen 2013 [17]	China	33914	60.1±13.2	6.81	1.43	1.16-1.77
Hamer 2011 [51]	UK	4338	62.9±9	3.04	1.52	1.01-2.30
Pan 2010 [22]	US	7415	50-75	9.88	1.29	1.18-1.40
O'Connor 2009 [52]	US	17076	40+	6.81	1.46	1.19-1.80
Almawi 2008 [53]	Bahrain	275	31-60	0.09	3.82	1.43-10.25
Golden 2008 [26]	US	5201	45-84	4.09	1.52	1.09-2.12
Luijendijk 2008 [54]	Netherlands	2931	61+	0.87	2.07	1.11-3.85
Maraldi 2007 [55]	Italy	2522	70-79	7.38	1.31	1.07-1.61
Engum 2007 [28]	Norway	37291	30-89	3.36	1.24	0.78-1.98
Engum 2007 [28]	Norway	37291	30-89	0.97	1.56	0.73-3.31
Kim 2006 [56]	South Korean	521	65+	1.39	1.00	0.40-2.50
de Jonge 2006 [57]	Spain	4803	55+	5.75	1.41	1.08-1.83
Brown 2006 [58]	Canada	88776	20-95	9.94	1.04	0.94-1.15
Polsky 2005 [59]	US	8387	51-61	8.30	1.17	0.98-1.41
Palinkas 2004 [35]	US	971	50-89	4.85	0.73	0.41-1.30
Bisschop 2004 [60]	Netherlands	1839	55-85	7.96	0.73	0.53-1.00
Rajala 1997 [61]	Finland	734	55	0.83	2.10	1.20-4.00
Wing 1990 [62]	US	64	30-70	2.84	1.96	0.70-2.05
Weyerer 1989 [63]	Hungary	1536	15+	0.39	3.15	1.69-5.87

### Publication bias

Funnel plot and Egger's test were employed to estimate the publication bias among the included studies. The funnel plot showed generally symmetrical distribution, suggesting no evidence of publication bias for the outcomes in both directions. Egger's test also found low possibility of publication bias (*p* = 0.262 for the direction of depression leading to diabetes and *p* = 0.326 for the direction of diabetes leading to depression).

## DISCUSSION

Diabetes and depression are two highly prevalent chronic diseases around the world, and increasing evidence indicates the high rates of co-occurrence of the two diseases. The present study was designed to conduct a quantitative assessment on the bidirectional relationships between the two diseases to provide directions for their prevention and treatment. It was found that there was a 34% increased risk of developing diabetes in depression patients than non-depressive subjects, and a 28% increased risk of developing depression in patients with diabetes compared with non-diabetic subjects. Thus, the study provides evidence that the relationships between diabetes and depression are bidirectional with similar strengths in both directions.

The mechanisms accounting for the relationships between diabetes and depression should be multiple in terms of biological, psychological and socioeconomic determinants [64–66]. First, depression can be regarded as an additional risk factor for diabetes. Depression patients are less likely to be compliant with dietary and weight loss recommendations and are more prone to be lack of physical exercise, which can lead to worsening of obesity and insulin resistance, and thus may increase the risk of developing diabetes. Some drugs used to treat depression may lead to weight gain and obesity, which could make individuals susceptible to the development of diabetes [67, 68]. Second, diabetes can also increase the risk of depression. For patients with diabetes, poor control of blood sugar, strict diet and physical exercise requirements, and treatment may increase the incidence of depression. It was found that chronic stress could cause hyper-activation of the hypothalamic-pituitary-adrenal axis and an increase in cortical, which has been proposed to be an important pathway to interpret the clinical relationships between diabetes and depression [69–71]. In addition, on the basis of the large-scale genome wide association studies about type 2 diabetes and major depressive disorder, we performed a bioinformatics analysis on the genetic overlap between the two diseases [72]. The overlapped single nucleotide polymorphisms and functional enrichment pathway of the annotated genes were identified, which provided clues for future treatment strategies for the two diseases [72].

Our analysis has several limitations. First, we analyzed the effect of gender on the risk of diabetes in patients with depression, while the current available data cannot permit us to perform the gender subgroup analysis for the direction of diabetes leading to depression. Second, as many studies did not specify the type of diabetes, we analyzed the overall relationships of diabetes with depression. Third, several case-control and cross-sectional studies were employed in the bidirectional analysis and thus we cannot exclude the recall and selection bias.

## MATERIALS AND METHODS

### Search strategy and selection criteria

With the combination of the following terms “depression” or “depressive” and “diabetes”, the Medline and PsycINFO databases were scanned for articles written in English and published in peer-reviewed journals up to October 2016. The conference proceedings and reference lists from retrieved articles were also screened to obtain additional relevant reports. Cohort, cross-sectional and case-control studies providing OR and 95%CI, or enough data to calculate the two parameters, of risk of developing diabetes in patients with depression or vice versa, were eligible for inclusion. Two investigators (QSZ and LS) independently screened the literature and extracted information from the eligible studies. Any disagreement was resolved by consensus. The flow chart in Figure 1 summarized the references selection process. In total, 51 studies were finally included in the bidirectional analysis [13–63], among which, 32 studies were about the risk of diabetes in patients with depression, and 24 studies were about the risk of depression in patients with diabetes.

### Meta-analytic methods

The meta-analysis was performed for the extracted fully adjusted ORs employing the Stata statistical software version 12.0 (Stata Corp LP, College Station, Texas) with the random effects model. To assess the effect of gender on the relationships between diabetes and depression, the gender-subgroup analysis was further performed. Heterogeneity was evaluated employing Q-test and *I^2^* score. Funnel plot and Egger's test were used to examine the publication bias among the included studies.

## CONCLUSIONS

In summary, the present quantitative assessmentprovides evidence that the relationships between diabetes and depression are bidirectional with similar strengths in both directions, which implies that physicians should be aware of the co-occurrence of depression and diabetes.
